# Chromosome-level genome assembly and annotation of the moon jellyfish *Aurelia coerulea*

**DOI:** 10.1038/s41597-026-07102-9

**Published:** 2026-04-10

**Authors:** Xinyue Hu, Yang Guo, Ze Zhang, Liyuan Wang, Duoyuan Chen, Zhenkun Zhuang, Minxiao Wang, Shiping Liu, Song Feng

**Affiliations:** 1 College of Life Sciences, University of Chinese Academy of Sciences, Beijing, 100049 China; 2 State Key Laboratory of Genome and Multi-omics Technologies, BGI Research, Hangzhou, 310030 China; 3 Key Laboratory of Spatial Omics of Zhejiang Province, BGI Research, Hangzhou, 310030 China; 4 Institute of Oceanology, Chinese Academy of Sciences, Qingdao, 266071 China; 5 Key Laboratory of Systems Health Science of Zhejiang Province, School of Life Science, Hangzhou Institute for Advanced Study, Hangzhou 310024; University of Chinese Academy of Sciences, Hangzhou, China

**Keywords:** Genome, Genomics, Genome informatics

## Abstract

*Aurelia coerulea* (moon jellyfish), a radially symmetrical metazoan in the phylum Cnidaria, possesses key features pertinent to understanding the evolutionary origins of nervous systems. Here, we employed a combination of long-read sequencing, short-read sequencing, and Hi-C chromatin conformation capture techniques to generate a chromosome-level assembly of the *A. coerulea* genome. The final assembly comprises approximately 554.10 Mb distributed across 21 chromosomes, achieving a scaffold N50 of 24.06 Mb and demonstrating high completeness (protein BUSCO score: 93.0%). Approximately 71.48% of the genome consists of transposable elements. We identified 26,777 protein-coding genes, of which approximately 72.28% have been functionally annotated. This chromosome-level genome provides an essential resource for elucidating early neural evolution and advancing our understanding of cnidarian biology.

## Background & Summary

The moon jellyfish (*Aurelia coerulea*) is a globally distributed scyphozoan species of ecological and evolutionary importance, serving as a dominant gelatinous zooplankton in coastal marine ecosystems. The moon jellyfish, characterized by a translucent saucer-shaped body, displays considerable size variation, typically ranging from about 10 centimeters to 30 centimeters in diameter. Highly adaptable, this species thrives across diverse marine environments, ranging from tropical to subarctic waters and from open ocean to brackish estuarine habitats^1^. *A. coerulea* reproduces both sexually and asexually. Asexual reproduction occurs either through strobilation budding or podocyst. Strobilation involves sequential developmental stages—from polyp to early strobila, late strobila, and finally ephyra. Budding allows new polyps to form directly from a parent polyp^2,3^. Podocyst was regarded as the dormant stage produced by polyps when suffering from adverse environmental conditions such as starvation.

*A. coerulea* plays a vital role as a model organism for studying cnidarian development, symbiosis, and environmental adaptation. And it also has been increasingly recognized as an important player in ecosystem functions and biogeochemical cycles^4,5^. Adult medusae, characterized by their translucent bell-shaped bodies and trailing tentacles armed with stinging cells (cnidocytes), exert profound impacts on marine food webs through predation on planktonic organisms such as copepods and fish larvae^6^. Population blooms of *A. coerulea*, generally considered to result from climate change and human disturbances to coasts (e.g., eutrophication, overfishing, habitat modification, agriculture, etc), profoundly affect ecosystems by disrupting fisheries, clogging power plant cooling systems, and altering planktonic food webs through increased predation on copepods and other microfauna^7^. Also, characterized by its complex medusoid phase, it serves as a crucial subject for genomic analyses aimed at understanding the evolution of morphological complexity in animals. Comparative studies with closely related species retaining only the simpler polyp stage (e.g., corals and sea anemones) highlight *Aurelia*’s significance. Its possession of specialized rhopalial sensory organs and adaptation to a pelagic predatory niche make it an outstanding model for elucidating the origins of nervous and sensory systems^3^.

Although several genomic studies have been conducted on jellyfish species, including species within the class Scyphozoa, a number of publicly available jellyfish genome assemblies, particularly those generated primarily from short read sequencing, remain fragmented. For instance, early draft scyphozoan assemblies such as *Aurelia aurita* (ARSv1.0; scaffold N50 0.18 Mb)^8^, *Nemopilema nomurai* (draft assembly; scaffold N50 2.71 Mb)^9^, and *Chrysaora quinquecirrha* (draft reference; scaffold N50 733.65 kb)^10^ illustrate that many available resources still lack chromosome level continuity. Recent genomic studies have further advanced scyphozoan genomics, including the chromosome level *A. coerulea* assembly reported by Dong *et al*.^11^, which generated an approximately 566 Mb genome, reported a diploid chromosome number of 2n = 44, and annotated 32,035 protein coding genes.

**Fig. 1:**
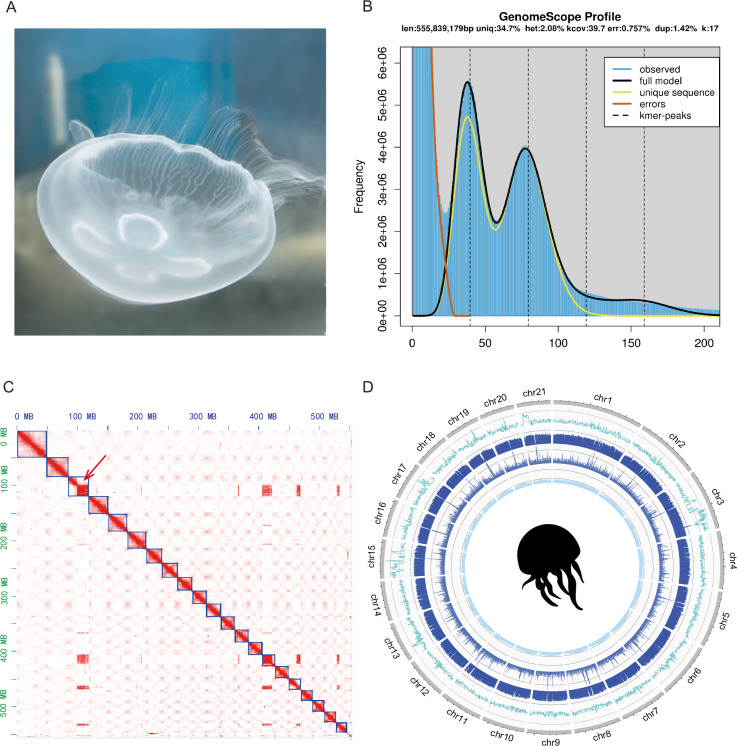
Genomic characteristics of *A. coerulea*. (**A**) Image of adult *A. coerulea*. (**B**) Genome size estimation using GenomeScope version 1.0 with 17-mer. (**C**) Genome-wide all-by-all Hi-C matrix. The red arrow on Chromosome 3 indicates a potential assembly discontinuity. Users should exercise caution when performing fine-scale downstream analyses in this specific genomic interval. (**D**) Circos view of the assembled chromosomes showing marker distributions at 2-Mb sliding windows from outer to inner circle: gene density, GC content, tandem repeat density, transposable element density.

However, for many cnidarian taxa, broader comparative analyses can still be limited by the quality and contiguity of currently available assemblies, which affects analyses of structural variation, repetitive elements, and synteny. To complement existing resources and to demonstrate a streamlined, reproducible assembly workflow, we present a chromosome-level genome assembly of *A. coerulea* generated using a combination of PacBio HiFi long-read sequencing, short-read whole-genome sequencing (WGS), and Hi-C chromatin conformation capture (Table 1). The assembled *A. coerulea* genome is 554.10 Mb in size with 37.37% GC content. After Hi-C scaffolding, 98.71% of the assembly was anchored to 21 pseudo-chromosomes (Tables 2, 3, Fig. 1). Transposable elements occupied 71.48% of the genome, of which long terminal repeat (LTR) elements account for 39.82% (Table 4). We predicted 26,777 protein-coding genes in the *A. coerulea* genome, and 72.28% of these genes can be functionally annotated using at least one public database (Table 5). The non-coding RNAs (ncRNAs) including tRNA, rRNA, miRNA, and snRNA, were annotated with a total length of 0.64 Mb (Table 6). In total, assessment of the final genome assembly itself using the metazoan Benchmarking Universal Single-Copy Orthologs (BUSCO) set revealed a completeness of 87.0% (C:87.0% [S:86.7%, D:0.3%], F:6.6%). When assessed on the predicted protein-coding gene set, the completeness reached 93.0% (C:93.0% [S:92.5%, D:0.5%], F:1.8%), indicating high quality of the gene annotation.

**Table 1:** Statistics of sequencing data.

Category	Reads(M)	Bases(Gb)	Depth(×)
WGS	2238.78	223.88	404.04
PacBio	1.77	33.37	60.22
Hi-C	637.82	95.67	172.67
RNA-seq	721.27	72.13	130.17

**Table 2:** Statistics of genome assembly.

Category	Number
Genome size (bp)	554,095,296
Number of contigs	149
Number of chromosome-scale sequences	21
Number of unplaced scaffolds	104
Contig N50 (bp)	20,249,868
Contig N75 (bp)	16,309,453
Scaffold N50 (bp)	24,061,078
GC content (%)	37.37
Number of genes	26,777
Average gene length (bp)	13,449
Genome BUSCO (BUSCO software)	C:87.0%[S:86.7%,D:0.3%],F:6.6%,M:6.4%,n:954
Protein BUSCO	C:93.0%[S:92.5%,D:0.5%],F:1.8%,M:5.2%,n:954
Transcriptome BUSCO	C:92.6%[S:92.2%,D:0.4%],F:2.4%,M:5.0%,n:954
Gene set Completeness (OMArk)	S:87.86%,D:3.2%[U:3.1%,E:0.09%],M:8.94%

**Table 3:** Statistics of Hi-C scaffolding.

Sequences	Length (bp)	Percentage (%)
Chr1	49,417,701	8.92%
Chr2	35,538,025	6.41%
Chr3	34,177,997	6.17%
Chr4	32,475,843	5.86%
Chr5	31,721,956	5.73%
Chr6	31,029,773	5.60%
Chr7	26,040,675	4.70%
Chr8	25,802,007	4.66%
Chr9	24,061,078	4.34%
Chr10	23,630,219	4.26%
Chr11	23,611,818	4.26%
Chr12	23,311,773	4.21%
Chr13	22,695,286	4.10%
Chr14	22,514,845	4.06%
Chr15	21,875,200	3.95%
Chr16	21,417,956	3.87%
Chr17	20,971,611	3.78%
Chr18	19,491,548	3.52%
Chr19	19,341,539	3.49%
Chr20	19,297,652	3.48%
Chr21	18,499,408	3.34%
Total length	546,923,910	98.71%
Unplaced scaffolds	7,171,386	1.29%

**Table 4:** Statistics of transposable elements (TE) annotation.

Type	Length(bp)	Percentage of genome(%)
DNA	2,556,700	0.44%
LINE	4,147,021	0.72%
SINE	441,123	0.08%
LTR	167,115,005	29.05%
Unclassified TEs	154,793,227	26.92%
Unknown	82,096,937	14.27%
Total	411,150,013	71.48%

Note: Percentages were calculated based on the total physical length of scaffolded chromosomes(575,209,453 bp), including gaps between anchored contigs.

**Table 5:** Statistics of gene functional annotation.

Category	Number	Percentage(%)
Total	26,777	/
Swissprot	9,687	36.18%
KEGG	6,502	24.28%
TrEMBL	16,832	62.86%
Interpro	10,785	40.28%
GO	6,232	23.27%
Overall	19,355	72.28%

**Table 6:** Statistics of ncRNA annotation.

Type		Number	Length (bp)	Percentage (%)
miRNAs		2	148	0.0001%
tRNAs		1643	244,825	0.0044%
rRNAs	rRNA	360	374,239	0.0675%
	18S	59	105,610	0.0191%
	28S	64	240,035	0.0433%
	5S	58	19,778	0.0036%
	5.8S	179	8,816	0.0015%
snRNA		10	21,609	0.0039%

Across core chromosome scale metrics, our assembly is broadly consistent with the previously published resource, supporting the robustness and reproducibility of chromosome level *A. coerulea* genome references. At the same time, differences in contig level contiguity and predicted gene number between the two assemblies are likely attributable to technical factors such as sequencing data characteristics, assembly and scaffolding decisions, and annotation pipelines rather than biological differences. We also note that minor differences in pseudo chromosome number among assemblies, such as 21 in our resource versus 22 reported previously, can arise from alternative merge and split decisions during Hi-C based scaffolding at ambiguous boundaries, and should be interpreted as assembly supported representations in the absence of cytogenetic validation. Taken together, the two assemblies provide complementary value. One offers stronger contig-level continuity, while the other is supported by deeper sequencing coverage. These high-quality genomic data of the *A. coerulea* represent a valuable resource for further studies on cnidarian phylogeny, genome evolution, and ecological adaptation.

## Methods

### Sampling and sequencing

Specimens of *A. coerulea* were collected in the Jiaozhou Bay, China (36.07°N, 120.16°E) and were cultivated in the Institute of Oceanology, Chinese Academy of Sciences. Samples were preserved in RNAlater immediate after sampling (Thermo Fisher Scientific) and stored at −80°C prior to nucleic acid extraction. Total genomic DNA was isolated from the tissue of a single individual using the QIAamp DNA Mini Kit (Qiagen) for subsequent whole-genome sequencing (WGS). For WGS, DNA fragmentation was performed using a Covaris E220 sonicator, followed by size selection of approximately 200 bp fragments with AMPure XP beads (Beckman). The selected fragments underwent eight cycles of PCR amplification and were sequenced on the DNBSEQ platform (BGI) in a paired-end 100 bp configuration (Table 1). High-molecular-weight (HMW) DNA for PacBio HiFi sequencing was obtained from the same individual used for WGS. The extraction followed a CTAB-based method optimized for HMW DNA preparation, including tissue grinding in liquid nitrogen and the final purification with AMPure XP beads (Beckman Coulter) to retain long fragments. The size distribution of DNA was verified using a Femto Pulse system (Agilent). Long-read sequencing was conducted on the PacBio Sequel II system. DNA quality was assessed using Qubit fluorometer (Thermo Fisher Scientific) and pulsed-field gel electrophoresis (BioRad). Subsequently, DNA was sheared to ~15 kb using g-TUBE (Covaris), followed by end-repair, and size-selected using BluePippin (Sage Science). One SMRT cell was sequenced in circular consensus sequencing (CCS) mode (Table 1). For Hi-C library construction, *A. coerulea* tissue was freeze-dried, powdered, resuspended in nuclei isolation buffer, and incubated in 0.5% SDS for 10 min at 62 °C. Nuclei were then collected by centrifugation, and nuclear DNA was digested using the restriction enzyme *Mbo*I (NEB). Digested fragments were subsequently end-filled, biotinylated, and ligated using T4 DNA ligase (NEB). Post-purification, DNA was sheared, biotinylated fragments were captured using Dynabeads MyOne Streptavidin T1 (Invitrogen), and the captured DNA was amplified and sequenced on the NovaSeq. 6000 platform (Illumina) in a paired-end 150 bp layout (Table 1). To facilitate genome annotation, RNA sequencing (RNA-seq) was performed. Total RNA extracted with TRIzol (Invitrogen) was reverse-transcribed into complementary DNA (cDNA) using HiscriptII (Vazyme). The resulting cDNA fragments were subjected to terminal repair, A-tailing, and connecting adaptors. Strand-specific library was purified with AMPure XP beads (Beckman Coulter) and sequenced on the DNBSEQ platform, generating 7.21 Gb of 100 bp paired-end data.

### Genome assembling and Hi-C scaffolding

A genome survey was conducted with WGS data using Jellyfish (v2.2.6)^12^ at K-mer 17, resulting in an estimated genome size of 555.84 Mb and heterozygosity of 2.08% for *A. coerulea*. The genome was assembled from PacBio HiFi long-read data utilizing hifiasm (v0.16.1) with parameters: -k 45 -r 2 -a 2 -m 2,000,000 -p 20,000 -l 0^13^. Following assembly, PacBio reads were realigned to the draft assembly using minimap2 (v2.14)^14^, and duplicated regions were identified and removed using Purge_Dups (v1.2.3) with default parameters. Potential contaminant contigs were detected by Kraken2^15^, and those classified as Bacteria were removed. The decontaminated contig-level assembly was assessed using BUSCO (v5.2.2)^16^ and Compleasm (v0.2.6)^17^ with metazoan odb10 (Table 2). The quality control of Hi-C data was performed using HiC-Pro v3.2^18^ (Table 1), after which contigs were scaffolded using 3D-DNA^19^. Assembled chromosomes were visualized and adjusted in Juicebox (v2.17)^20^, and 98.71% of the contigs were anchored to 21 chromosomes (Table 3, Fig. 1C). The final assembly is 554.10 Mb with a scaffold N50 length of 24.06 Mb (Table 2, Fig. 1D).

### Repeat annotation

Repetitive sequences were comprehensively annotated using a combination of de novo, homology-based, and structural searches. Tandem repeats were identified with Tandem Repeats Finder (v4.09.1) using a MaxPeriod of 2000^21^. For transposable elements (TEs), a de novo repeat library was first constructed by integrating outputs from LTR_Finder (v1.0.6, parameter “-C”)^22^ and RepeatModeler (v1.0.8, default parameters)^23^, the latter of which incorporates RECON and RepeatScout. This custom library was then combined with the curated Repbase (v21.01)^24^ database to create a non-redundant master library. The entire genome was screened against this master library using RepeatMasker (v4.0.6)^25^ with parameters “-nolow -norna -no_is”. Finally, annotations from all sources were merged, and overlaps were resolved to generate a consolidated set of repetitive elements. The final statistics are summarized in Table 4.

### Gene prediction and non-coding RNA annotation

Protein-coding genes were annotated through an integrated pipeline combining *ab initio* prediction, homology-based approaches, and transcriptomic evidence. Augustus (v3.1)^26^ was utilized for *ab initio* gene prediction. Homology-based annotation involved aligning gene sets from four closely related jellyfish species (*A. aurita atlantic*^8^, *Rhopilema esculentum*^27^, *Chrysaora quinquecirrha*^28^, and *Morbakka virulenta*^8^) to the *A. coerulea* genome using Blast (v2.2.26)^29^. Alignment hits were linked to candidate gene regions using GenBlastA^30^, and gene models were subsequently predicted using GeneWise (v2.2.0)^31^ based on candidate gene sequences and their flanking 2-kb genomic regions. Additionally, RNA-seq data were mapped to the genome assembly using HISAT (v2.1.0)^32^. Transcriptomic evidence for annotation was subsequently generated using StringTie (v1.3.4)^32^, and potential coding regions were identified with TransDecoder (v5.7.1) (github.com/TransDecoder/TransDecoder). Results from the three prediction methods were integrated using EvidenceModeler (EVM) (v1.1.1)^33^ with parameters set to --segmentSize 5000000 --overlapSize 200000. The integration weights assigned were: AUGUSTUS 1, GeneWise 3, and transdecoder 10. All annotated protein-coding genes were functionally annotated by searching against the public databases, including Swiss-Prot (v201709), KEGG (v87.0), InterPro (v55.0), and TrEMBL (v201709) (Table 5). Completeness of the predicted gene set was assessed using BUSCO (v5.2.2)^16^ (Table 2), and the gene annotation quality was assessed using OMArk (v0.3.0)^34^.

Non-coding RNAs (ncRNAs), including transfer RNAs (tRNAs), ribosomal RNAs (rRNAs), small nuclear RNAs (snRNAs), and microRNAs (miRNAs), were predicted in the genome assembly. tRNAs were identified using tRNAscan-SE (v1.3.1)^35^ with default parameters. Invertebrate rRNA sequences were aligned against the genome assembly using barrnap (v0.9) (https://github.com/tseemann/barrnap) software with its database of eukaryotes. For miRNA and snRNA annotation, candidate regions were first identified by aligning the genome assembly against the Rfam database (v14.1)^36^ using BLAST software^29^ (e-value = 1). Subsequently, snRNAs and miRNAs were annotated using INFERNAL (v1.1.1)^37^ with default parameters (Table 6).

## Data Records

All genomic and transcriptomic datasets generated for *A. coerulea* are hosted across the NCBI and Figshare repositories. The final chromosome-level genome assembly is accessible via the NCBI Assembly database under accession JBPOCX000000000^38^. Supporting this assembly, all raw sequencing reads are archived in the NCBI Sequence Read Archive (SRA) under the study accession SRP596850^39^ (associated with BioProject PRJNA1284069), which encompasses data from WGS (SRX29512917), PacBio HiFi (SRX29505274), Hi-C (SRX29497767), and four biological replicates of RNA-seq (SRX29505275–SRX29505278). Additional curated genomic resources are provided through a dedicated Figshare^40^ repository. This repository includes the genome assembly sequences in FASTA format (AcoGenome.fasta) and the standardized structural gene models in GFF format (AcoGenome.gff).

## Technical Validation

Sequencing was performed on a PacBio library, which yielded long reads with an N50 of 17.7 kb, providing approximately 60× coverage. The assembled genome size was 554.10 Mb, consistent with the 555 Mb estimate from Jellyfish. Assembly accuracy was high, evidenced by a Merqury (v1.3)^41^ quality value of 61.35. The assembly comprised 149 contigs exhibiting an N50 of 20.2 Mb and an N75 of 16.3 Mb. Hi-C data processing yielded a valid read rate of 19.36%, and subsequent scaffolding successfully anchored 98.71% of contigs onto 21 chromosomes. Whole-genome sequencing (WGS) reads were aligned to the final genome assembly using BWA MEM (v0.7.17) (github.com/lh3/bwa), and the mapping rate calculated by samtools (v1.9)^42^ (flagstat, excluding secondary mappings) was 98.15%. Furthermore, PacBio HiFi reads aligned with minimap2 (v2.17)^14^ (“-ax asm20”) achieved a 92.37% mapping rate. BUSCO analysis (v5.2.2)^16^ in protein identified 904 out of 954 BUSCOs (887 complete), corresponding to a completeness of 93.0%. Additionally, Compleasm (v0.2.6)^17^ estimated the genome completeness at 92.56%. Gene annotation quality, assessed using OMArk (v0.3.0)^34^, showed a completeness of 91.06%.

## Data Availability

All sequencing data, including WGS, PacBio, Hi-C, RNA-seq, as well as the assembly (JBPOCX000000000)^38^ have been deposited at the NCBI (National Centre for Biotechnology Information) repository under project PRJNA1284069, SRP596850^39^. The genome assembly and annotations of *A. coerulea* are also available at Figshare^40^.

## Code availability

The parameters of the software were default. No specific script was used in this work.
